# Immune-modulating therapy with granulocyte-macrophage colony-stimulating factor (GM-CSF) in refractory rhino-orbital-cerebral mucormycosis – a case report

**DOI:** 10.3389/fimmu.2025.1620545

**Published:** 2025-09-17

**Authors:** Piremiya Thayanantham, Evan Kalin-Hajdu, Simon Dufresne, Philippe J. Dufresne, Julien Viau-Lapointe, Jan-Alexis Tremblay

**Affiliations:** ^1^ Université de Montréal, Montréal, QC, Canada; ^2^ University ophtalmology center, Hôpital Maisonneuve-Rosemont, Montréal, QC, Canada; ^3^ Division of Infectious Diseases and Medical Microbiology, Department of Medicine, Hôpital Maisonneuve-Rosemont, Montréal, QC, Canada; ^4^ Laboratoire de santé publique du Québec, Institut national de santé publique du Québec, Sainte-Anne-de-Bellevue, QC, Canada; ^5^ Division of Critical Care Medicine, Department of Medicine, Hôpital Maisonneuve-Rosemont, Montréal, QC, Canada

**Keywords:** mucormycosis, GM-CSF, immunomodulation, mucorales, case report

## Abstract

This report outlines the case of a woman with rhino-orbital-cerebral mucormycosis following diabetic ketoacidosis, refractory to systemic and local antifungal treatment as well as repeated extensive sinonasal debridement. Adjunctive Granulocyte-Macrophage Colony-Stimulating Factor (GM-CSF) therapy was associated with significant clinical improvement. This treatment restored visual acuity, and the excellent outcomes were maintained at the 24-month follow-up. This is the first report of successful use of a short course (5 days) of GM-CSF for refractory rhino-orbital cerebral mucormycosis in an adult patient after diabetic ketoacidosis, highlighting the therapeutic potential of this pragmatic approach.

## Introduction

Mucormycosis is an invasive fungal infection caused by Mucorales fungi (1). These opportunistic pathogens primarily affect individuals with compromised immunity, such as those with uncontrolled diabetes, hematologic malignancies, organ transplants, or those on immunosuppressive therapies (2). Prompt extensive sinonasal debridement, antifungal treatment and reversal of underlying immunosuppression represents the backbone of management (3). Despite optimal care, patients with severe mucormycosis infection face dismal outcomes, with mortality rates ranging from 70 to 90% (1, 4). Early recognition is paramount, as delayed diagnosis is associated with increased mortality (5).

Diabetic ketoacidosis is a well-established risk factor for mucormycosis, primarily because of impairment of chemotaxis and phagocytic functions in myeloid cells under conditions of hyperglycemia and acidosis (6). Elevated levels of free ferric iron also contribute to the proliferation of Mucorales species. Furthermore, critically ill patients with a severe infection can develop secondary immune dysfunction, a state of exhaustion of the immune system with profoundly dysregulated host responses (7). This immune dysfunction may contribute to progression of mucormycosis, even after the underlying triggers have been addressed and appropriate antimicrobial and surgical interventions have been implemented (8).

Granulocyte-Macrophage Colony-Stimulating Factor (GM-CSF) is an immunomodulatory cytokine produced by various circulating and tissue-resident immune cells. It promotes the maturation and phagocytic functions of granulocytes and monocytes. Adjunctive immunotherapy with GM-CSF can stimulate the immune system in critically ill patients and has been previously reported as successful in various severe fungal infections (9).

We report the use of GM-CSF as an adjunct therapy in the management of a severe, refractory case of rhino-orbital cerebral mucormycosis after diabetic ketoacidosis.

## Case description

**Figure 1:**
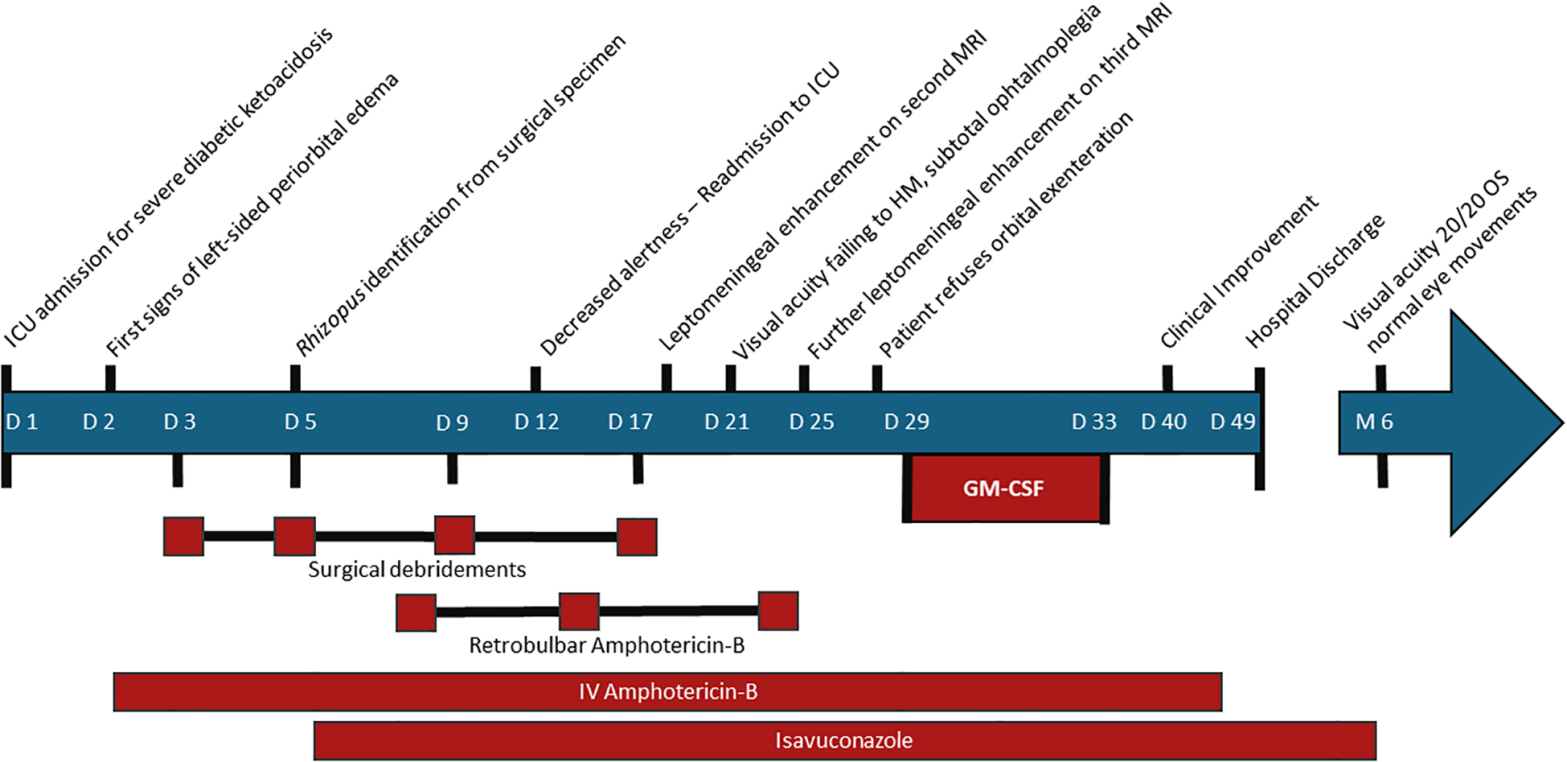
Timeline of clinical events and interventions in a case of severe rhino-orbital cerebral mucormycosis. ICU, Intensive Care Unit; MRI, Magnetic Resonance Imaging; HM, Hand Motion.

A 51-year-old female with no significant past medical history presented with severe diabetic ketoacidosis (DKA) as the initial manifestation of latent autoimmune diabetes (LADA). After her ketoacidosis was corrected on day 2 of admission, she developed acute left periorbital edema, along with pain and erythema (**Figure 1**). She was admitted to the intensive care unit (ICU).

Initial evaluation and Computed Tomography (CT) imaging suggested orbital cellulitis and sinusitis. She was started on broad-spectrum antibiotics and empirical intravenous liposomal amphotericin B at a daily dose of 5 mg/kg. Endoscopic sinus surgery was performed, and tissue samples were collected for microbiological analysis.

**Figure 2:**
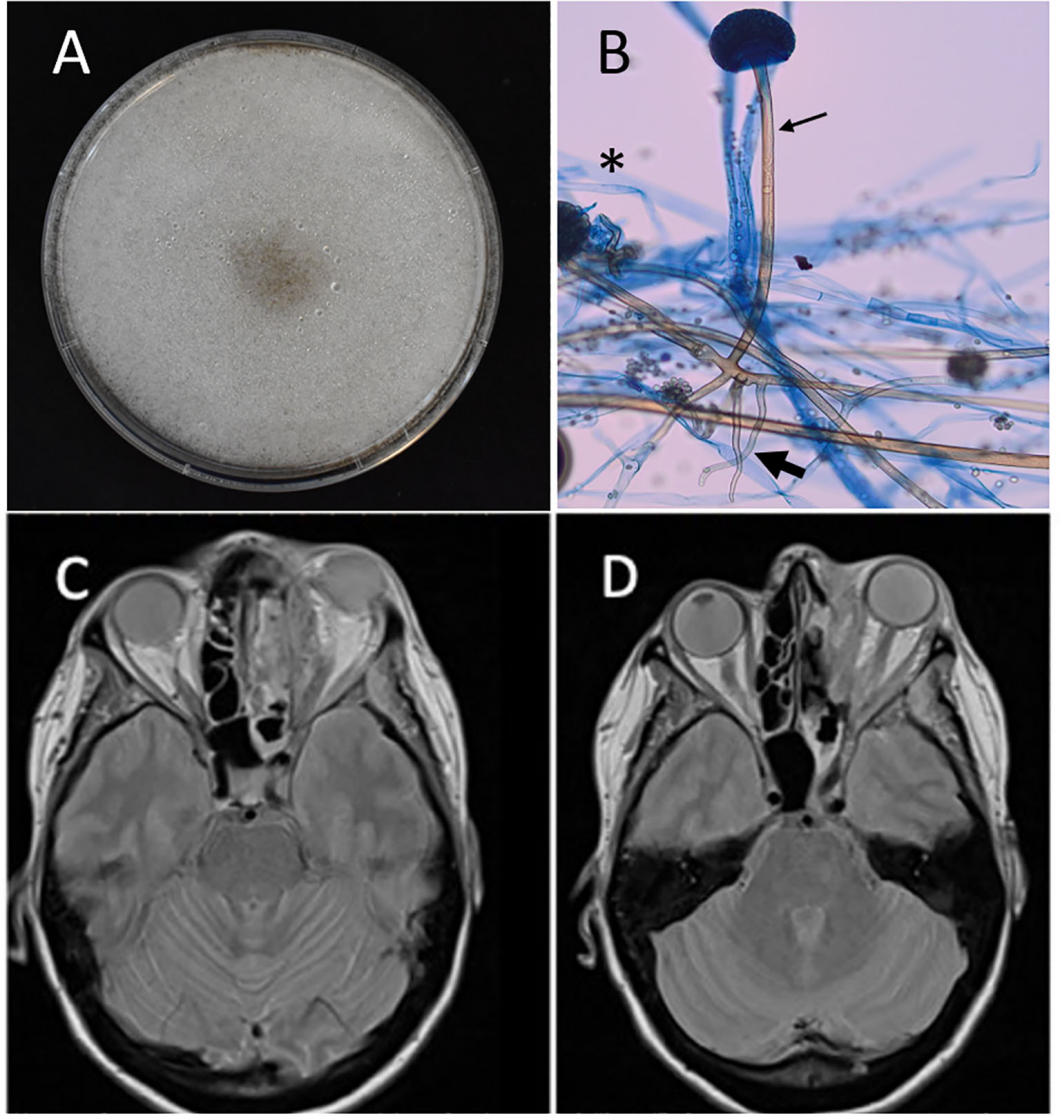
**(A)** Macroscopic appearance of the isolate, subcultured on Potato Dextrose Agar and grown for 3 days at 30°C, showing a rapidly growing, woolly, grayish brown colony typical of Mucoralean fungi. **(B)** Microscopic features of the isolate using lactophenol cotton blue preparation (at 400X magnification), showing large ribbon-like pauciseptate hyphae (asterisk sign), unbranched sporangiosphore with lack of apophysis under sporangia (thin arrow) and large rhizoids (bold arrow). **(C, D)** MRI findings on day 18 of severe rhino-orbital cerebral mucormycosis. T2 signal hyperdense abnormalities of the optic nerve secondary to ischemic neuropathy. Perineural extension with infiltration of the orbital apex, the orbital fissure, and the cavernous sinus.

Direct microscopic examination with calcofluor white stain revealed non-septate hyphae consistent with Mucorales species, with cultures demonstrating colony morphology and microscopic features consistent with *Rhizopus* species (**Figure 2**). Genomic sequence-based analysis was performed at our reference laboratory and identified the species as *Rhizopus delemar* (**Supplementary Material S1**, sequences deposited as GenBank accession records PX026267 and PX026291). Antifungal susceptibility testing with broth microdilution revealed high minimal inhibitory concentrations (MIC) to isavuconazole and posaconazole (>8 mg/l and >16 mg/l, respectively), and lower MIC to amphotericin B (1 mg/l).

The patient’s condition rapidly deteriorated. She developed decreased alertness and worsening periorbital swelling. Repeat CT showed increased retroseptal infiltration and over the next two weeks, the patient continued to decline, with increasing edema and reduced eye movements. Intravenous isavuconazole was added at a dose of 200 mg intravenously every 8 hours, and intravenous liposomal amphotericin B was increased to 10 mg/kg/day. Retrobulbar liposomal amphotericin B (3 mg) was also administered daily for 3 days by the ophthalmology team.

Despite tight glycemic control, optimal antifungal treatment, and extensive sinonasal endoscopic debridement on days 5, 9, and 17, the second Magnetic Resonance Imaging (MRI) on day 18 after admission revealed worsening optic nerve involvement and leptomeningeal enhancement (**Figure 2**). The patient’s condition continued to decline, with visual acuity dropping to hand motion in the left eye, limitations in extraocular movements, and further central nervous system involvement noted on repeat imaging the following week.

Exenteration was discussed as a potential option, but the patient refused any further surgical intervention. Repeat retrobulbar liposomal amphotericin B injections were also considered but were deemed inappropriate due to worsening orbital edema and chemosis.

Due to the patient’s lack of response to combined antifungal therapy, refusal of further surgery and overall poor prognosis, the decision was made to offer immune adjuvant therapy. Granulocyte-Macrophage Colony-Stimulating Factor (GM-CSF, sargramostim/leukine, Partner Therapeutics Inc) was introduced at a dose of 250 mcg (1 ml) subcutaneously daily for 5 days (days 29 to 33 after admission). No fever or other adverse events were noted.

One week following GM-CSF treatment, the patient showed significant clinical improvement, with reduction in pain and edema of the periorbital region and stabilization of central nervous system involvement on MRI. No adverse effects were noted. Two weeks after completing GM-CSF treatment and 49 days after admission, the patient was discharged successfully, with a 6-month course of oral isavuconazole. She declined to undergo subsequent control MRIs at follow-up. Two years after discharge, her ophthalmologic evaluation was normal with 20/20 visual acuity, normal eye movements and no residual proptosis.

## Discussion

Mucormycosis is a highly morbid and often fatal fungal infection. Incidence of mucormycosis has been rising in the last few years, possibly due to increased awareness, more frequent use of immunosuppressive drugs and, recently, the COVID pandemic (10). Rhino-orbital cerebral involvement is mostly observed in individuals with poorly controlled diabetes mellitus, particularly during ketoacidosis (11). In contrast, patients with hematological malignancies and organ transplant recipients often present with pulmonary involvement and disseminated infections (2). *Rhizopus arrhizus* (formerly *Rhizopus oryzae*) is one of the most common agents of mucormycosis worldwide (12, 13). *Rhizopus delemar* is considered a variant of this species (*R. arrhizus* var. *delemar*), although some experts argue it represents a distinct species. Its epidemiology and specific virulence remain poorly defined due to the limited availability of species-level identification in most mucormycosis cases (14). While its taxonomic classification remains under debate, multiple reports have shown that this species or variant exhibits high MICs for posaconazole and isavuconazole (15) as seen with our isolate.

Management principles for rhino-orbital mucormycosis include prompt extensive sinonasal debridement and antifungal therapy, mainly with liposomal amphotericin B (AmB). Posaconazole and isavuconazole are recommended as salvage therapy in patients who cannot be treated with AmB. Despite the paucity of prospective clinical data, antifungal combination therapy (amphotericin B + posaconazole or isavuconazole) is widely used (3). Extensive repetitive surgical debridement of all necrotic sinonasal tissue is also necessary to halt the progression of mucormycosis and increase the effectiveness of antifungal therapy. Exenteration of infected orbital tissue has not been shown to improve survival, but is still suggested if orbital necrosis is present on imaging (16). In contrast, when the orbit remains vitalized, there has been an increasing trend towards retrobulbar AmB with promising results (16). However, even with these combined medical and surgical interventions, mortality rates for rhino-orbital cerebral mucormycosis remain as high as 62%, with little progress in improving survival over the past two decades (4).

GM-CSF has been described as a potent strategy in multiple retrospective studies of patients with severe refractory infections, with seemingly good outcomes notably in patients with severe fungal infections (9). A recent systematic review (17) highlights the potential role of immune-adjuvant therapy with GM-CSF in invasive fungal diseases, with a reported overall response rate of 82% in 65 published cases. Of these, 13 patients received GM-CSF for an infection caused by Mucorales species, of which 6 had specifically a rhino-orbital-cerebral infection. Interestingly, all these cases underwent longer duration of GM-CSF treatment, ranging from 20 days to as long as 7 months. The presented case is, to our knowledge, the first to show a clinical response after a shorter course (5 days) of treatment, which emphasizes its potential role as a pragmatic and feasible strategy for such patients. All these findings suggest that GM-CSF can help restore granulocyte and myeloid cell function, which can tip the immune host response towards staving off and eventually clearing off the infection completely, in a context of underlying immune exhaustion.

While the patient’s significant improvement following GM-CSF treatment is noteworthy, it’s important to recognize that we cannot definitively determine how much GM-CSF contributed to her recovery, due to the interplay of various treatment modalities and the timing of administration. Further studies are needed to confirm its role in the management of severe mucormycosis.

## Conclusion

This is the first report of successful use of a short course of GM-CSF for refractory rhino-orbital cerebral mucormycosis in an adult patient after diabetic ketoacidosis. Leveraging the host response with immunomodulatory therapies like GM-CSF shows promise as an adjunct to antifungal and surgical management for severe, refractory mucormycosis.

As this is a single case report, additional data from larger studies are essential to better understand the role of GM-CSF in the management of such cases. Future strategies for improving mucormycosis management should involve a multifaceted approach, targeting both the pathogen’s virulence mechanisms and enhance the host’s immune capabilities, thereby opening new therapeutic avenues for these vulnerable patients.

## Patient perspective

At last follow-up, after the patient was asked for her written informed consent to describe her case, she expressed her gratitude towards the team and her enthusiasm for the GM-CSF treatment she received at a moment of great incertitude regarding her own survival. She specifically asked us to publish her case to make this therapy known and to encourage further research on immune adjuvant treatment in mucormycosis infections.

## Data Availability

The original contributions presented in the study are included in the article/**Supplementary Material**. Further inquiries can be directed to the corresponding author.
