# Supplementary material for: Immune-modulating therapy with granulocyte-macrophage colony-stimulating factor (GM-CSF) in refractory rhino-orbital-cerebral mucormycosis – a case report
Source: Front Immunol. 2025 Sep 17;16:1620545. doi: 10.3389/fimmu.2025.1620545 (PMC12484159; doi:10.3389/fimmu.2025.1620545)
Supplement: Supplementary file 1 [file Table1.docx]

Supplementary Material S1

**Specific methodology for genomic identification of *Rhizopus delemar* specimen**

Genomic sequence-based identification was performed at our reference center (Laboratoire de santé publique du Québec) by amplicon Sanger sequencing of the ITS (using ITS1 and ITS4 primers) and D1/D2 (NL1 and NL4 primers) regions of ribosomal DNA according to standard protocol [1].

Sequences were aligned against comprehensive sequence dataset for Mucorales species from NCBI Refseq taxonomic studies [2, 3].

Best matching species was found to be *Rhizopus delemar* with 100% ITS and D1D2 sequence homology (CBS 392.95 type strain; AB181326 and JN939196) followed by *Rhizopus arrhizus* with 99.7 to 99.3% similarity (CBS 112.07 type strain; JN206323 and HM849659).

Sequences were deposited as GenBank accession records PX026267 and PX026291.

References:

1. CLSI. *Interpretive Criteria for Identification of Bacteria and Fungi by DNA Target Sequencing. 2^nd^* ed*.* CLSI guideline MM18. Wayne, PA: Clinical and Laboratory Standards Institute. 2018.

2. Walther, G., et al., *DNA barcoding in Mucorales: an inventory of biodiversity.* Persoonia, 2013. **30**: p. 11-47.

3. Wagner, L., et al., *A new species concept for the clinically relevant Mucor circinelloides complex.* Persoonia, 2020. **44**: p. 67-97.
